# Compatibility of personal care thickening polymers in sulphate‐free surfactants

**DOI:** 10.1111/ics.70065

**Published:** 2025-12-25

**Authors:** Tobias Halthur, Jonas Carlstedt, Marta Gubitosi, Debora W. Chang, Eric S. Johnson

**Affiliations:** ^1^ CR Competence AB Lund Sweden; ^2^ Biomedical Science, Faculty of Health and Society Malmö University Malmö Sweden; ^3^ Biofilms – Research Center for Biointerfaces (BRCB) Malmö University Malmö Sweden; ^4^ The Procter & Gamble Company Cincinnati Ohio USA

**Keywords:** formulation/stability, miscibility, phase separation, polymers, salt levels, surfactants, thickening

## Abstract

**Objective:**

Formulators today know that when traditional surfactants and polymers are replaced new problems occur. What was before a ‘thickening problem’ becomes a ‘miscibility problem’: the ingredients do not play well with each other anymore. However, the scientific rules are not different for these systems, which can also be understood and controlled. This study was therefore made, focusing on one of the important challenges, namely the phase separation and miscibility problem of polymers and surfactants in aqueous solution in ratios and concentrations of interest as shampoo chassis.

There are three main reasons for these problems: (1) Impurities and variability from raw materials, (2) by‐products or excess components and (3) high levels of background salt remaining from processing. In this work, the third factor has been investigated to learn when salts should be reduced or counteracted depending on the polymer type used for enabling thickening in the new formula spaces. Thus, the study is fundamental in nature but aims to facilitate communication with suppliers of both surfactants and polymers as well as with colleague formulators. It aims at an increased holistic approach where all ingredients are acknowledged constituents of the system, and even minor additions of simple salts are noted as relevant variables to control to maximize product performance.

**Methods:**

Visual characterization of phase behaviour and viscosity assessment has been performed where three polymers have been combined with two different sulphate‐free surfactants. Emphasis has been put on the effect of background salts.

**Results:**

The levels of background salts affect both viscosity and compatibility of the systems investigated, however, to different extents depending on the nature of the materials. It is therefore important to control the levels of salt (often included in the raw materials), and how this affects the nature of the polymer–surfactant interactions, to reach the desired formulation attributes.

**Conclusion:**

While prior knowledge based on classical aqueous polymer–surfactant systems was seen to be valid also for newer sulphate‐free systems (as long as the salt concentration could be controlled), fully compatible mixtures where viscosity and surfactant concentrations truly meet the levels desired for the end formulation proved difficult to find.

## Graphical abstract

Phase behaviour for alpha olefin sulfonate surfactant 10–15 wt% combined with 1 wt% sodium carboxy methyl cellulose, clearly indicating the sensitivity in miscibility when adding various levels of background salt.

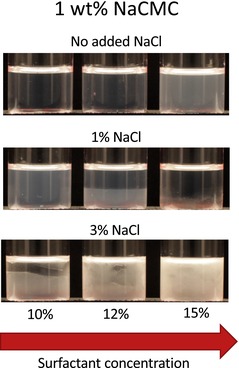

## Résumé

**Objectif:**

Les formulateurs savent aujourd’hui que le remplacement des tensioactifs et polymères classiques entraîne de nouveaux problèmes. Ce qui était auparavant un « problème d’épaississement » devient un « problème de miscibilité » : les ingrédients ne sont plus compatibles entre eux. Cependant, les règles scientifiques ne sont pas différentes pour ces systèmes, qui peuvent également être compris et contrôlés. Cette étude a donc été réalisée en se concentrant sur l’un des défis importants, à savoir le problème de la séparation des phases et de la miscibilité des polymères et des tensioactifs en solution aqueuse dans des proportions et des concentrations intéressantes pour les bases de shampooing.

Il existe trois raisons principales à ces problèmes : (1) La présence d’impuretés et la variabilité des matières premières, (2) les sous‐produits ou les composants en excès, et (3) les taux élevés de sels résiduels provenant du traitement. Dans le cadre de ce travail, le troisième facteur a été étudié afin de déterminer quand les sels doivent être réduits ou neutralisés en fonction du type de polymère utilisé pour permettre l’épaississement dans les nouveaux espaces de formulation. Cette étude est donc de nature fondamentale, mais elle vise à faciliter la communication avec les fournisseurs de tensioactifs et de polymères ainsi qu’avec les collègues formulateurs. Elle vise à adopter une approche plus générale, dans laquelle tous les ingrédients sont reconnus comme des constituants du système, et où même des ajouts mineurs de sels simples sont considérés comme des variables pertinentes à contrôler afin d’optimiser les performances du produit.

**Méthodes:**

Une caractérisation visuelle du comportement des phases et une évaluation de la viscosité ont été réalisées en combinant trois polymères avec deux tensioactifs sans sulfate différents. L’accent a été mis sur l’effet des sels résiduels.

**Résultats:**

Les taux de sels résiduels influent à la fois sur la viscosité et sur la compatibilité des systèmes étudiés, mais à des degrés différents selon la nature des matériaux. Il est donc important de contrôler les taux de sels (souvent présents dans les matières premières) et leur incidence sur la nature des interactions polymères‐tensioactifs afin d’obtenir les propriétés souhaitées pour la formulation.

**Conclusion:**

Si les connaissances antérieures basées sur les systèmes classiques de polymères‐tensioactifs en solution aqueuse se sont avérées valables également pour les nouveaux systèmes sans sulfate (à condition que la concentration en sels puisse être contrôlée), il a été difficile de trouver des mélanges entièrement compatibles dont la viscosité et les concentrations en tensioactifs correspondaient réellement aux taux souhaités pour la formulation finale.

## INTRODUCTION

Surfactants have been combined with polymers for different reasons in many types of water‐based formulations. Regardless of formulation, the role of the surfactants is generally to provide wetting, emulsification, solubilization and/or cleaning. Polymers, on the other hand, are often added not only to improve bulk properties, such as to stabilize a dispersion, hinder redeposition of dirt or to modify the viscosity, but also to provide surface properties such as deposition or for surface modification (either by itself or as coacervates with the surfactant).

It has long been known that surfactants and polymers may interact with each other to various extents depending on the nature of both polymer and the surfactant system selected. One key fundamental is to understand that when mixing surfactants with a polymer, depending on the interactions, you can either have miscibility or phase separation [1, 2]. In general, miscibility is reached at dilute conditions (for at least one of the components), or when the interactions between the surfactant and the polymer are moderately attractive, while phase separation is expected for strong interactions and can either be associative (when the attraction is too strong) [3, 4, 5] or segregative (for repulsive interaction) [6]. However, segregation is also expected for systems where the interactions are nonexistent or very weak [7, 8]. The main interactions at play here are electrostatic (attractive or repulsive) and hydrophobic (always attractive), with smaller contributions from van der Waals interactions and hydrogen bonding, and the magnitude of the net interaction can to some extent be estimated by determining the CAC (critical aggregation/association concentration), which indicates if the surfactant is self‐associating on or in close proximity to the polymer at concentrations lower than the CMC. These ‘aggregates’ are usually micelle‐like and can either be induced by the polymer [9], or formed as mixed micelles for the case where the polymer has hydrophobic modifications that can be part of the micelle core [2]. In addition, when at least one of the species involved carries a charge, the extent as well as the nature of net interaction is greatly affected by the presence of counterions and background simple salts. For instance, it has been shown that a system with oppositely charged polymer and surfactants can go from strong attraction and associative phase separation, to weak attractive and miscible, to non‐attractive with segregation just by increasing the background salt level [10, 11]. Segregation with added salt is then reached both due to the fact that the electrostatic attraction is shielded but is also enhanced by the fact that many surfactant systems are prone to micellar growth with added salt, which essentially leads to a system of two macromolecules with very little attraction and segregative phase separation as a consequence [1].

Over the years, several polymer–surfactant combinations have been identified, optimized and extensively used to deliver useful performance benefits for products such as shampoos, hard surface cleaners, laundry detergents and fabric softeners. However, there are new demands on the fast‐moving consumer goods industry to identify and utilize greener materials, which has brought new challenges to fully develop polymer and surfactant combinations that work together synergistically to drive performance rather than causing stability failures such as phase separation.

Replacing historically used naturally derived or synthetic petrolatum‐based surfactants, and to some extent polymers, to newer greener biobased, bio‐sourced or even biosurfactants introduces several issues that can be related to impurities. These impurities can generally be categorized in three groups: impurities and variability from raw materials (such as large variations in lipid chain length and degree of saturation, extracts from bioprocessing, etc.), by‐products or excess residuals (alcohol isomers, free fatty acids, etc.) and high levels of background salt remaining from processing. Each of these groups have the potential, in different ways, to affect the surfactant–polymer compatibility as well as the overall performance of the surfactant in various environments.

In this paper, we are focusing on the effect of high salt levels in new sulphate‐free surfactant materials and how that affects polymer compatibility, miscibility and the end performance in terms of the ability to build viscosity.

For this purpose, we have selected two commercially available sulphate‐free anionic surfactants (which are used in personal care products already on the market). The surfactants used were Diapon HF‐SF which is a short chain (10 carbon) Methyl Taurate surfactant (C10MT), which is not expected to give micellar growth, and the longer (14–16 carbon) BioTerge AS‐40 HP, which is an alpha olefin sulfonate (AOS) surfactant capable of micellar growth under the right circumstances. These surfactants were combined with three chemically modified biopolymers, with specific different characteristics for comparison. Hydroxy ethyl cellulose (HEC) was selected as a hydrophilic non‐ionic model polymer, while a hydrophobically modified HEC (HM‐HEC) was selected as a hydrophobic non‐ionic model polymer. Finally, sodium carboxy methyl cellulose (Na‐CMC) was selected as an anionic model polymer. All these surfactant and polymer materials are used extensively in personal care formulations and were selected from a set of polymers previously assessed (as received) at Procter & Gamble as potential thickeners in surfactant systems which are historically difficult to thicken with salt, such as those containing methyl taurate or AOS. In addition, carrageenan, algin, diutan gum, modified starch and xanthan gum were also previously evaluated at Procter & Gamble. The majority of these polymers either failed to thicken in the presence of surfactants or even thinned out upon the addition of surfactants. Furthermore, the few polymers that provided initial thickening in the presence of surfactants either displayed long‐term instability characterized by phase separation or provided thickening but with an undesirable, lumpy texture. Therefore, there is a need for robust and efficient thickening and suspending technologies that are more biodegradable and compatible with anionic surfactant‐based cleansers used in the areas of personal cleansing and hair care, while still being reliably, sustainably and responsibly sourced. The aim of this study was therefore to see if compatibility can be achieved by controlling the background salt levels through purification and controlled additions. The ambition is to test the established understanding of polymer surfactant compatibility in this new context while at the same time provide first principle guidance to formulators as well as ingredient producers.

## MATERIALS AND METHODS

### Materials

Sodium methyl cocoyl taurate (C10MT), available under the trade name Diapon™ HF‐SF and sodium C14–C16 olefin sulfonate (AOS), available under the trade name BioTerge® AS‐40 HP, was sourced from NOF Corporation (Japan) and Stepan Company (USA), respectively. Sodium chloride (NaCl), ACS reagent grade and methanol hypergrade for LC–MS were both purchased from Merck (Germany). HEC, available under the trade name Natrosol 250™, and cetyl hydroxyethylcellulose (HM‐HEC), available under the trade name Natrosol™ 330 CS/Polysurf 67 CS were sourced from Ashland Inc. (USA), while Na‐CMC, available under the trade name Walocel™ CRT 40000 GA with a degree of substitution (DS) of 0.9, was sourced from Dow Chemical Company (USA). The dialysis membrane used was a Spectra/Por® 7 MWCO 1000, 45MM from Repligen Corporation (USA), and deionized water was used for all material purification protocols as well as for preparation of all samples.

### Purification of materials

Prior to sample preparation, both surfactants were purified by recrystallization while all three polymers were purified by dialysis; the protocols are detailed below.

For C10MT purification, equal volumes of the as received Diapon™ HF‐SF solution (24–30 wt% active) and methanol (800 mL each) were mixed to precipitate the C10MT. The mixture was left standing at room temperature without stirring for approximately 1 day while crystals were forming. The solids were then separated from the liquid by filtration using a Büchner funnel with filter paper and vacuum. The solid surfactant was collected, dried at 40°C, and sent to an analytical lab (ALS Scandinavia AB, Luleå, Sweden) for elemental analysis together with a reference sample of dried Diapon™ HF‐SF. For AOS purification, 2 L methanol and 1.5 L of the as received BioTerge® AS‐40 HP solution (75% of the water phase), and 0.5 L water (25% of the water phase) were mixed and left standing at room temperature without stirring for approximately 1 day while crystals were forming. The solids were then separated from the liquid by filtration using a Büchner funnel with filter paper and vacuum. The solid surfactant was collected, dried at 40°C and sent to an analytical lab (ALS Scandinavia AB, Luleå, Sweden) for elemental analysis together with a reference sample of dried BioTerge® AS‐40 HP.

Each different polymer was prepared as a 2 wt% aqueous solution, and each solution was placed in 50 mL plastic centrifuge tubes with a dialysis membrane placed on top. The membrane was held in place with a plastic cap with a hole. Dialysis was performed against deionized water for 5 days (deionized water was changed three times during the dialysis) and conductivity measurements on the polymer solutions were performed before and after dialysis on a 712 Conductometer from Metrohm Nordic AB (Sweden). After dialysis, each different polymer solution was lyophilized in a DW6‐85 freeze dryer from Heto‐Holten A/S (Denmark) for 24 h.

### Sample preparation and visual inspection

Samples of various combinations of surfactant, polymer and NaCl were prepared in 10 mL clear glass vials with a plastic cap by weighing the solids and pipetting the water to reach the desired weight per cent (wt%) of each individual component. Samples were vortexed and, if needed, placed on a sample rocker for several days, centrifuged for 25 min at 1000 RCF (Sigma K615 centrifuge) to collect all material at the bottom of the vials, and finally left on the lab bench for equilibration at room temperature for approximately 2 weeks before evaluation. Phase behaviour was evaluated visually, and the samples were examined in both normal and cross‐polarized light (photos taken for documentation), where the latter is used for detection of optical birefringence (samples observed both at rest and agitated) present in anisotropic phases. Additionally, a qualitative assessment of viscosity with the range ‘low’, ‘low medium’, ‘medium’, ‘medium high’, ‘high’ (desired viscosity for shampoo formulations), ‘very high’ and ‘stiff gel’ was performed, where ‘low’ corresponds to water like, ‘high’ corresponds to most shampoo formulations, and ‘very high’ is almost like a gel but still flows.

## RESULTS

### Purification

Elemental analysis indicated that the sodium and chloride content was reduced for both surfactants after purification through recrystallization. The reduction in sodium and chloride roughly corresponded to a reduction in NaCl from 2.5 to 0.5 wt% for C10MT and from 1.3 to 0.2 wt% for AOS. Conductivity measurements of the polymers indicated a reduction in background salt corresponding to an NaCl reduction from 3.2 to 1.2 wt% for HEC and from 1.9 to 0.9 wt% for HM‐HEC. Conductivity measurements were inconclusive for the Na‐CMC due to the high conductivity introduced by the charged polymer and its counter ions.

### Neat samples

As can be seen in Table 1, very little differentiation in phase behaviour is observed for the neat surfactant samples. The C10MT surfactant only exhibits low viscosity regardless of concentration and salt content and is only seen to be salted out at the highest salt concentration. The AOS, on the other hand, is partially salted out at 5 wt% NaCl and is also seen to increase in viscosity and to show shear induced birefringence indicating wormlike micelles both when increasing salt content (from 2 to 3 wt%) and when increasing surfactant concentration (from 5 to 15 wt% at 3 wt% NaCl).

**TABLE 1:** Visually assessed viscosity and phase behaviour for the neat C10MT (left) and AOS (right) surfactants at various concentrations and varying levels of added sodium chloride. Colours (viscosity rating), patterns and symbols (appearance assessment) are explained below the table. Please note that the colour in the appearance legend just exemplifies a viscosity, while the symbols and patterns are of interest here.

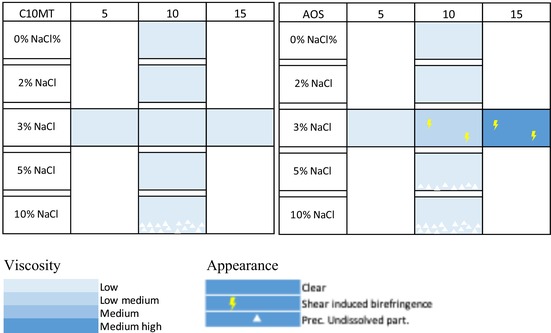

### Hydroxy ethyl cellulose

**FIGURE 1:**
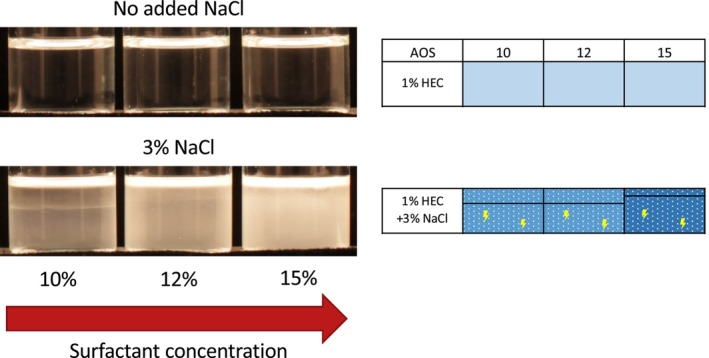
Example image of what some samples look like, in this case for AOS surfactant 10–15 wt% combined with 1 wt% hydroxy ethyl cellulose (HEC) with and without added salt. Data corresponding to rows 1 and 3 in Table 3, as illustrated by the inserted partial tables. Samples are illuminated from below and the top shiny part is the meniscus.

As can be seen in Tables 2 and 3, the non‐ionic polymer is compatible with both anionic surfactants even at the highest surfactant concentrations in the absence of added salt. On the other hand, the viscosity only shifts from low to medium when increasing concentrations, indicating that very little texture is built. When salt is added, phase separation is observed in both polymer–surfactant systems. For the highest concentrations of C10MT turbid stiff gels are formed in the bottom of the vials, leaving a low viscosity clear solution on top, whereas all three high concentrations tested for the AOS separate in two turbid phases where the bottom phase displays shear induced birefringence indicating wormlike micelles. In this case, the viscosity ranges from medium high to very high with increasing concentrations but is similar for top and bottom phases. Some sample images are shown in Figure 1.

**TABLE 2:** Visually assessed viscosity and phase behaviour for the C10MT surfactant at various concentrations combined with HEC at 1 and 2 wt% with and without added sodium chloride. Colours (viscosity rating), patterns and symbols (appearance assessment) are explained below the table, where desired outcome is highlighted in red. Please note that the colour in the appearance legend just exemplifies a viscosity, while the symbols and patterns are of interest here.

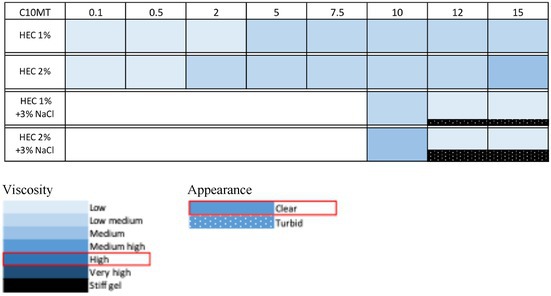

**TABLE 3:** Visually assessed viscosity and phase behaviour for the AOS surfactant at various concentrations combined with HEC at 1 and 2 wt% with and without added sodium chloride. Colours (viscosity rating), patterns and symbols (appearance assessment) are explained below the table, where desired outcome is highlighted in red. Please note that the colour in the appearance legend just exemplifies a viscosity, whereas the symbols and patterns are of interest here.

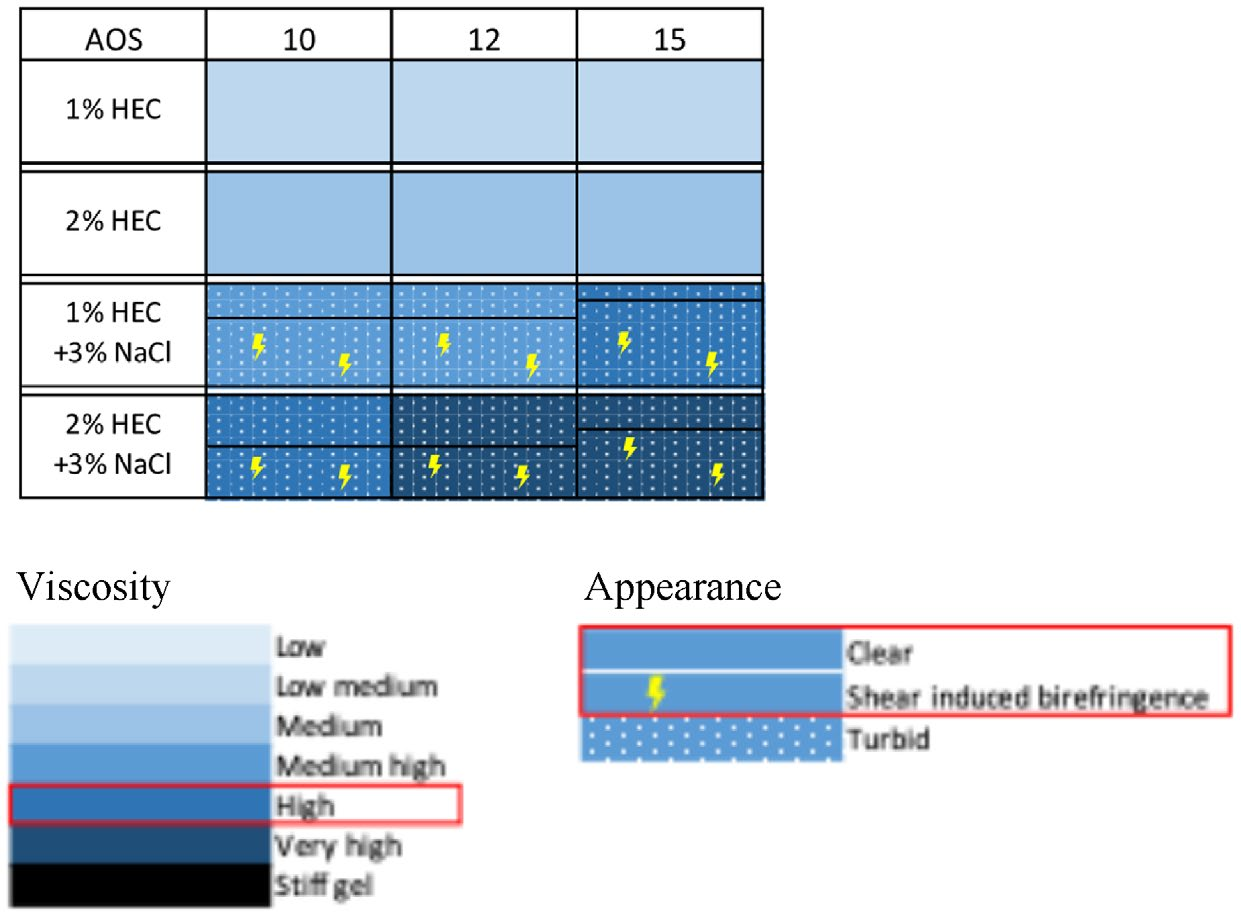

### Hydrophobically modified hydroxy ethyl cellulose (HM‐HEC)

The visually assessed compatibility was in general high when combining the HM‐HEC with C10MT at all concentrations investigated as seen in Table 4. Samples were slightly blueish and translucent but not turbid, and the translucence was observed to decrease with the surfactant concentration. Viscosity was highest at low surfactant concentration peaking slightly at 0.5 wt%.

**TABLE 4:** Visually assessed viscosity and phase behaviour for the C10MT surfactant at various concentrations combined with HM‐HEC at 1 and 2 wt%. Colours (viscosity rating), patterns and symbols (appearance assessment) are explained below the table, where desired outcome is highlighted in red. Please note that the colour in the appearance legend just exemplifies a viscosity, whereas the symbols and patterns are of interest here.

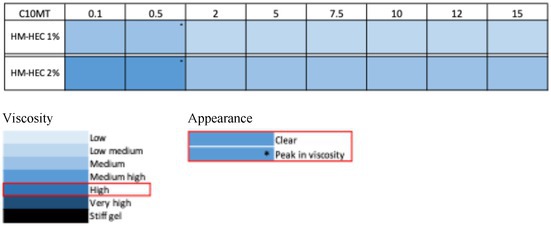

**FIGURE 2:**
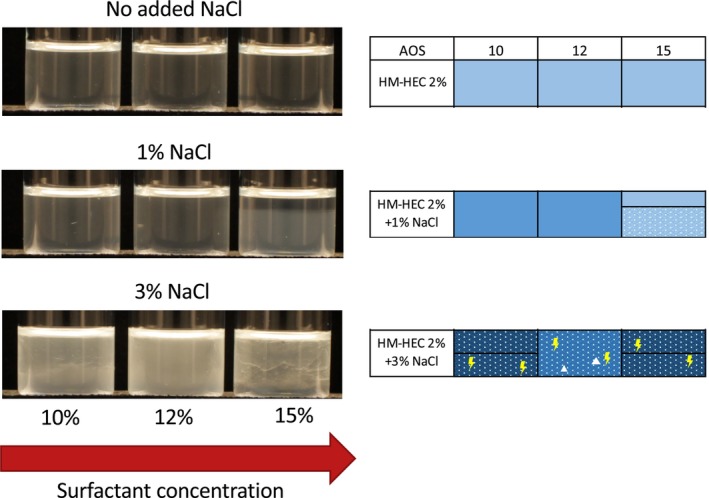
Example image of what some samples look like, in this case for AOS surfactant 10%–15% combined with 2% hydrophobically modified hydroxy ethyl cellulose (HM‐HEC). Images correspond to the rightmost three columns in Table 5, as illustrated by the inserted partial tables. Samples are illuminated from below and the top shiny part is the meniscus.

HM‐HEC also proved to be highly compatible with AOS at all concentrations studied as seen in top row of Table 5. In this case, viscosity was also noted to be very high at low surfactant concentrations at 2 wt% polymer, and an even slightly higher viscosity was maintained up to 7.5 wt% surfactant. Adding 1 wt% salt further extended this higher viscosity regime to 12 wt% surfactant before phase separation was observed at 15 wt%. With 3 wt% added salt phase separation was the norm at all high concentrations tested. Some sample images are shown in Figure 2.

**TABLE 5:** Visually assessed viscosity and phase behaviour for the AOS surfactant at various concentrations combined with 2 wt% hydrophobically modified hydroxy ethyl cellulose (HM‐HEC) with and without additions of sodium chloride. Colours (viscosity rating), patterns and symbols (appearance assessment) are explained below the table, where desired outcome is highlighted in red. Please note that the colour in the appearance legend just exemplifies a viscosity, whereas the symbols and patterns are of interest here.

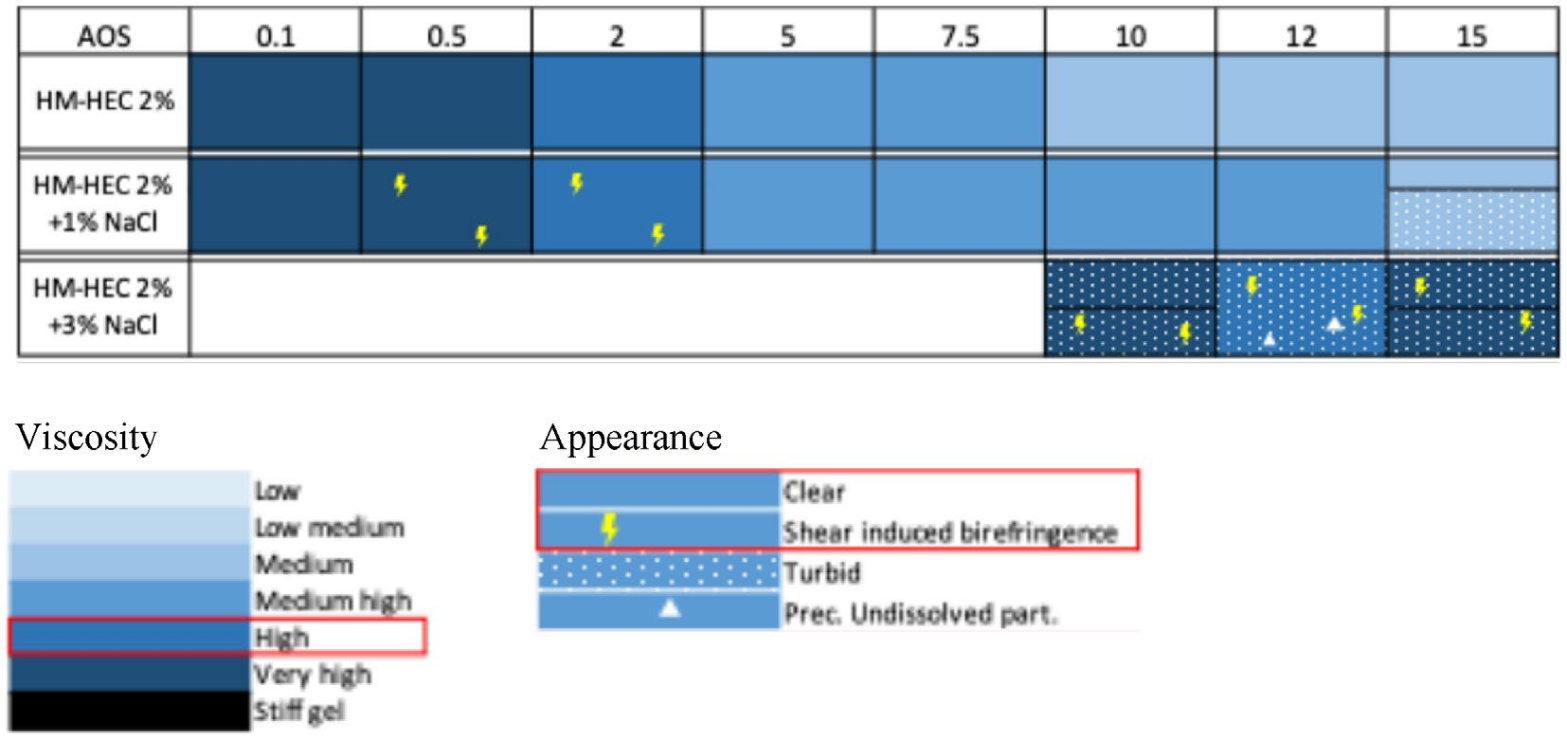

### Sodium carboxy methyl cellulose (Na‐CMC)

**FIGURE 3:**
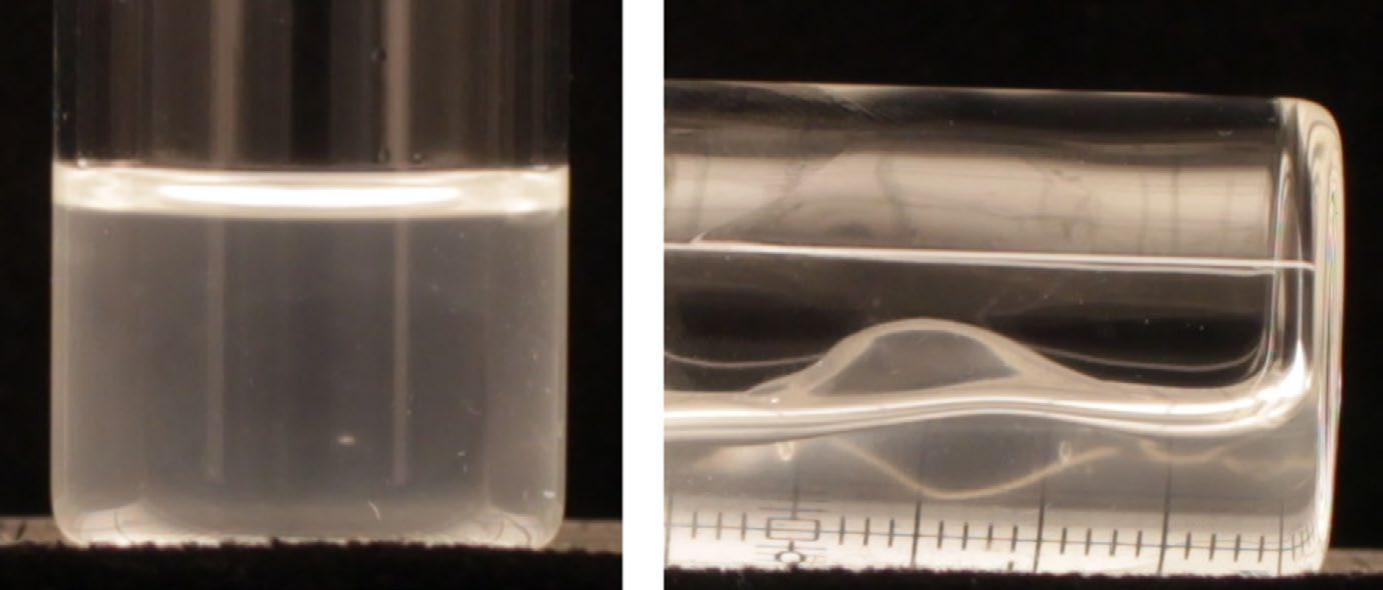
Example image of what a ‘blob’ can look like. Same sample standing up (left), and laying down (right), where the blob is more easily seen. Sample corresponds to 2 wt% Na‐CMC, 15 wt% C10MT and 2 wt% NaCl in Table 7.

The C10MT surfactant showed good compatibility with the same charge polymer Na‐CMC in the absence of added salt (see Tables 6 and 7). Viscosity was assessed as medium at 1 wt% polymer regardless of the surfactant concentration used but increased significantly up to high and even very high with shear induced birefringence for samples containing 2 wt% polymer. Shear‐induced birefringence in 2 wt% Na‐CMC samples is found at very low surfactant concentration which shows that in this specific case, the birefringence is a consequence of aligning polymers in the shear flow. Adding 2 or 5 wt% sodium chloride, however, generated phase separation by formation of gel ‘blobs’ at both polymer concentrations. These ‘blobs’ are very difficult to see due to matched refractive index to surrounding liquid (see Figure 3). The most extreme sample (2 polymer and 15 wt% surfactant) is macroscopically phase separated, with a clear medium‐viscosity top phase, and a turbid, birefringent bottom phase with shear induced birefringence, ‘blobs’, and medium‐high viscosity.

**TABLE 6:** Visually assessed viscosity and phase behaviour for the C10MT surfactant at various concentrations combined with sodium carboxy methyl cellulose (Na‐CMC) at 1 wt% with and without added sodium chloride. Colours (viscosity rating), patterns and symbols (appearance assessment) are explained below the table, where desired outcome is highlighted in red. Please note that the colour in the appearance legend just exemplifies a viscosity, whereas the symbols and patterns are of interest here.

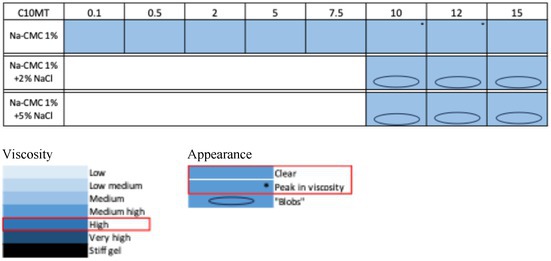

**TABLE 7:** Visually assessed viscosity and phase behaviour for the C10MT surfactant at various concentrations combined with Na‐CMC at 2 wt% with and without added sodium chloride. Colours (viscosity rating), patterns and symbols (appearance assessment) are explained below the table, where desired outcome is highlighted in red. Please note that the colour in the appearance legend just exemplifies a viscosity, whereas the symbols and patterns are of interest here.

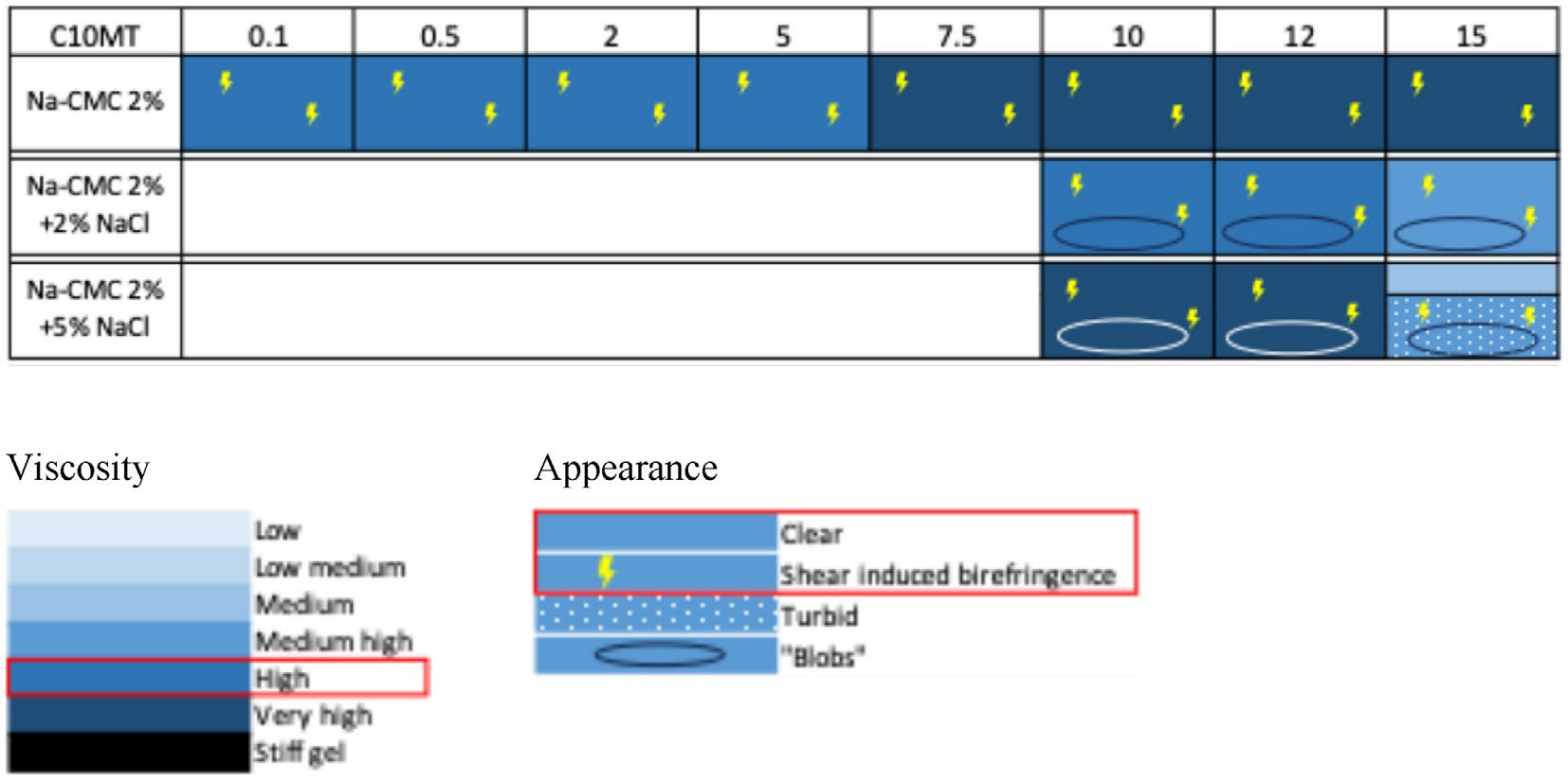

**FIGURE 4:**
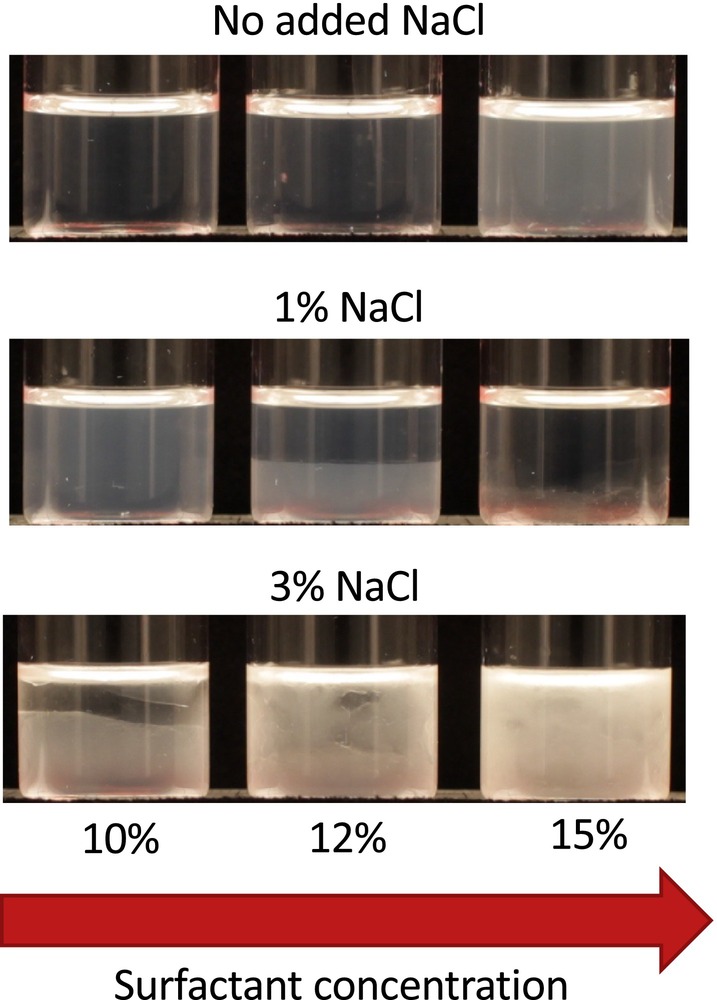
Example image of what some samples look like, in this case for AOS surfactant 10 to 15 wt% combined with 1 wt% NaCMC with and without added salt. Data corresponding to Table 8.

The anionic Na‐CMC polymer proved to be more sensitive when combined with the slightly longer tailed AOS surfactant, compared to the C10MT surfactant. This is seen by the fact that turbid samples with gel ‘blobs’ are formed even without added salt for the highest surfactant concentration in samples with 1 wt% polymer (see Table 8). The increased drive for phase separation is further substantiated when adding NaCl, where macroscopic phase separation and precipitation is observed with 12 wt% surfactant when only 1 wt% salt is added, and already at 10 wt% surfactant with 3 wt% salt, as seen in Table 8 and Figure 4. Increasing the polymer concentration to 2 wt% again led to higher viscosity but also to even more incompatibility and macroscopic phase separations (see Table 9).

**TABLE 8:** Visually assessed viscosity and phase behaviour for the AOS surfactant at various concentrations combined with Na‐CMC at 1 wt% with and without added sodium chloride. Colours (viscosity rating), patterns and symbols (appearance assessment) are explained below the table, where desired outcome is highlighted in red. Please note that the colour in the appearance legend just exemplifies a viscosity, whereas the symbols and patterns are of interest here.

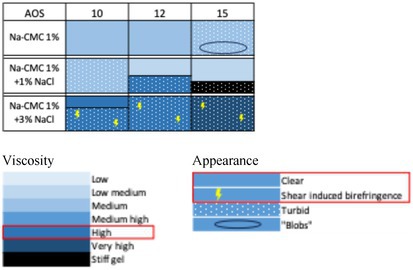

**TABLE 9:** Visually assessed viscosity and phase behaviour for the AOS surfactant at various concentrations combined with Na‐CMC at 2 wt% with and without added sodium chloride. Colours (viscosity rating), patterns and symbols (appearance assessment) are explained below the table, where desired outcome is highlighted in red. Please note that the colour in the appearance legend just exemplifies a viscosity, whereas the symbols and patterns are of interest here.

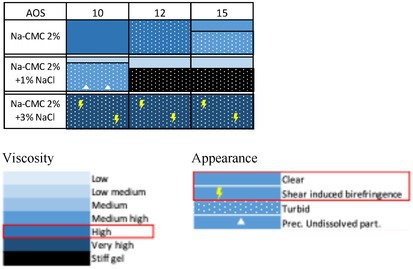

## DISCUSSION

### Neat surfactant samples

As seen in the results in Table 1, the shorter chained C10MT surfactant displayed high solubility and salt compatibility, sustaining full solubility at 10 wt% with 5 wt% added salt. On the other hand, it does not thicken with salt, nor increased concentration due to its inability to form elongated wormlike micelles. Looking at the structure of AOS with its longer tail (C14/C16 mixture), one would expect this surfactant to be able to form elongated wormlike micelles. This has not been seen in the lab and thus co‐surfactants, such as cocoyl amidopropyl betaine, combined with added simple salt are needed for inducing micellar growth to build viscosity. The purified version of AOS used in this study, however, clearly shows signs of forming elongated wormlike micelles at 10 wt% with 3 wt% added salt. This is strongly indicated by the increased viscosity and shear induced birefringence when shaking the sample (see Table 1). The viscosity is further increased when increasing the concentration to 15 wt% AOS with 3 wt% salt, but further increasing the salt content to 5 wt% leads instead to salting out and precipitation. The differences comparing this purified AOS and the as received material is a topic on its own as in addition to removing simple salts, it has been proven that alcohol isomers are also removed, that is, present in at least one commercial AOS. The specific study on AOS thickening will be presented in detail in a separate publication which is now in writing expanding the topic of this publication to one of the other causes of incompatibility mentioned in the introduction: presence of by‐products or excess residuals.

### Compatibility with HEC

When combining an anionic surfactant with a non‐ionic polymer one would expect a moderate attraction with micellization induced by the polymer at the CAC, where the micelles associated with the polymer impart charge to the polymer which can even lead to increased solubility and cloud point, and as a result good compatibility is to be expected [12, 13]. As seen in Table 2, C10MT is indeed compatible with HEC in the full concentration range examined both at 1 and 2 wt% polymer, and viscosity is seen to increase (although modestly) with both surfactant and polymer concentration. Similarly, the AOS is seen to exhibit perfect miscibility for both 1 and 2 wt% HEC all the way up to 15 wt% surfactant (see Table 2). However, both systems prove to be sensitive to additions of salt as phase separation is observed when 3 wt% salt is added. In the C10MT system a stiff turbid gel separates out with a clear low viscosity top phase for samples containing 1 or 2 wt% HEC with 12 or 15 wt% C10MT. The AOS system proves to be even more sensitive, where phase separation is noticed already at 10 wt% surfactant (both for 1 and 2 wt% HEC), but in this case, both phases are turbid, and the bottom phase displays shear‐induced birefringence indicating wormlike micelles. The incompatibility and dramatic change with additions of 3 wt% salt is even more obvious when looking at the samples as presented in Figure 1, where the simple mixture is seen to turn from well behaved with complete miscibility without salt, to messy and clearly not suitable for formulation with only 3 wt% of NaCl. Although catastrophic for a formulator, this behaviour can be predicted for systems where a charged surfactant is mixed with a non‐ionic polymer. This is due to that the electrostatic repulsion between head groups opposing micellization is reduced when adding salt, thus reducing the CMC and therefore also reducing the gain for polymer induced micellization. In other words, reduced attraction between surfactant and polymer and eventually elimination of the CAC. At the same time, the increased ionic strength also leads to that the ionic micelles are screened and consequently behave more like non‐ionic micelles, meaning that the system is now more similar to a non‐ionic–non‐ionic mixture where segregative phase separation is expected [1, 12]. In many ways, micelles can be viewed as macromolecules, and this situation is thus similar to a mixture of non‐ionic polymers where incompatibility and segregative phase separation is more or less the norm [1]. Segregation of non‐ionic systems is also known to increase with the molecular weight of the species, which also means that micellar growth will further increase the risk of phase separation. The latter consequence is most likely why phase separation is enhanced when switching to AOS which has a tendency for growth, which is also indicated by the induced birefringence seen in the phase separated samples [8]. Segregation in non‐ionic systems has also been discussed in terms of a depletion flocculation mechanism [14]. However, this entropic model is deemed unlikely due to the fact that it neglects short‐range interactions and thus gives rise to discrepancies between experimental results and model predictions. Furthermore, this model exaggerates the depletion effect due to relative size differences between surfactant micelles and the polymer radius of gyration [8].

To sum up, the non‐ionic HEC is compatible with both anionic surfactants studied at concentrations commonly used for personal care products. On the other hand, the resulting viscosity is only low or moderate and the systems are very sensitive to additions of salt most likely leading to segregative phase separation.

### Compatibility with HM‐HEC

**FIGURE 5:**
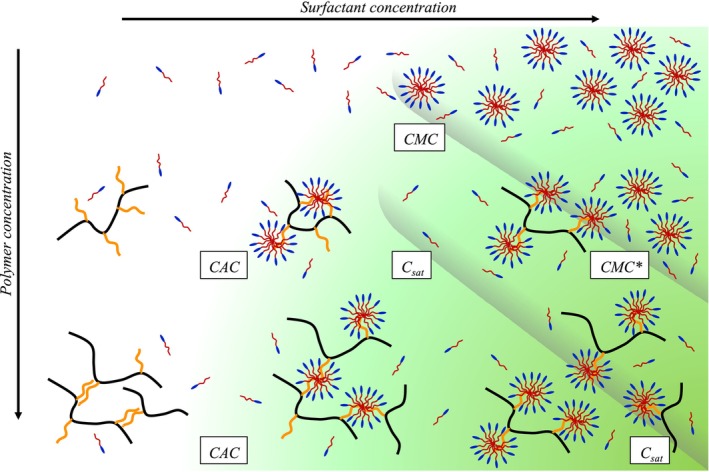
Schematic illustration of how surfactant micellization, polymer association and surfactant–polymer mixed micellization occurs in a system where hydrophobically modified polymers are mixed with surfactants at various concentrations and ratios. The number of micelles increases with increasing surfactant concentration as well as with the polymer concentrations (as depicted by the intensity of green) due to that they are formed at lower surfactant concentration as mixed micelles on the polymer when present. However, the number of free micelles decreases with increasing polymer concentration since surfactant added will continue to form micelles on the polymer until saturation is reached (*C*_sat_) and eventually an apparent *CMC* in presence of polymer (CMC*) is reached.

When a hydrophobic modification is introduced by, for example, being grafted to the backbone, polymer–surfactant interactions are mainly governed by hydrophobic interactions giving a low CAC which is commonly described as a mixed micellization situation [2]. In principle, this means that micelles are formed incorporating the hydrophobic modification as part of the micelle core well below the CMC of the surfactant as illustrated in Figure 5.

**FIGURE 6:**
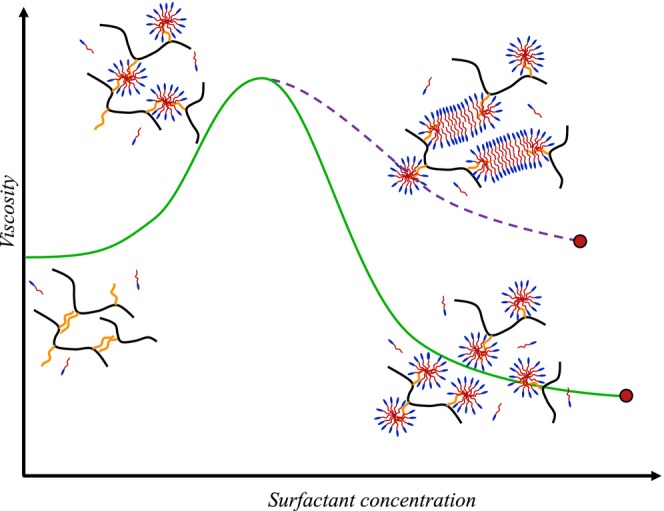
Schematic illustration of how viscosity changes with surfactant concentration when mixed with a hydrophobically modified polymer.

As presented, hydrophobically modified polymers often associate to each other if the concentration is high enough, creating a network and increasing the viscosity [2, 15], which is why they are often referred to as associative thickeners. This means that they can be efficient in building viscosity without surfactant (bottom left in Figure 5). When a low concentration of surfactant is added, mixed micelles starts to form at the CAC (mid‐section in the bottom of Figure 5), which can actually strengthen, or increase the lifetime of the polymer–polymer association, thus often increasing the viscosity by orders of magnitude [15, 16] which is further illustrated in the left side of Figure 6. This behaviour is indeed well reflected in the C10MT data presented in Table 4, where viscosity shifts from medium to medium‐high at 0.1 wt% surfactant when increasing the polymer concentration from 1 to 2 wt% (i.e. increasing the polymer‐polymer associations at higher polymer concentration). A small peak in viscosity is observed for both polymer concentrations when increasing the surfactant concentration from 0.1 to 0.5 wt%. As the surfactant concentration is further increased however, the viscosity is often reduced due to that the polymer‐polymer associations are broken in the presence of more micelles, as eventually there is one micelle per hydrophobic modification and the polymer is saturated [15, 16] (see bottom right in Figure 5), which is also most likely why we see a clear drop in viscosity when the C10MT concentration is further increased (see Table 4). The viscosity at high surfactant concentration is often lower than the viscosity built by polymer associations alone [15], visualized by the green line in Figure 6.

In theory, one way to extend the high viscosity regime to higher surfactant concentrations would be to maintain the optimum number of micelles formed (ideally one per polymer‐polymer association). This could either be done by switching to a surfactant with higher CMC and CAC (shifting the optimum to higher surfactant concentration), or by increasing the aggregation number in the micelles formed by transition from spherical micelles to vesicles [2] or to elongated micelles. There are examples in the literature where wormlike micelles have been combined with hydrophobically modified polymers [17], or with hydrophobically end‐capped polymers [18, 19] where both scenarios describe a high viscosity network system where wormlike micelles are ‘crosslinked’ with the hydrophobically modified polymer.

Thus, in principle, inducing micellar growth would broaden the formulation window and extend the high viscosity regime as visualized by the purple dashed line and illustration in Figure 6. In reality, this is a very difficult balance to maintain and can only be done with a surfactant prone to micellar growth, which is what was investigated using the AOS surfactant presented in Table 5. As seen, the compatibility is great at 2 wt% polymer for the whole concentration range of AOS tested without added salt. In this case, very high viscosity was observed already at low AOS concentrations, which is reduced to high at 2 wt%, medium high at 5 wt% and finally to down to only medium viscosity at 10 wt% surfactant concentration and higher, indicating that polymer–polymer associations and a viscosity building network is maintained to higher surfactant concentrations even without added salt. Adding 1 wt% salt to the system further extends the medium–high viscosity regime to 12 wt% surfactant, indicating that micellar growth is present and supports the structure. At 15 wt% surfactant, the sample phase separates, and samples with 3 wt% added salt are very turbid and phase separated at all high concentrations as seen in Table 5 and Figure 2. At this point, it is likely that all electrostatic interactions have been screened, and the situation compares to two non‐ionic macromolecule species (polymer with mixed micelles and elongated free micelles) that simply do not mix.

### Compatibility with Na‐CMC

When combining a polymer and a surfactant of the same charge, electrostatic repulsion, no CAC, and consequently, segregative phase separation is to be expected [1, 12, 20]. However, as for all systems, miscibility can be found when at least one of the components has low concentration [7]. Interestingly, though in our experiments, salt‐free purified C10MT shows miscibility in the full concentration range for both 1 and 2 wt% Na‐CMC, and at 2 wt% polymer, the highly sought high to very high viscosity suitable for personal care products can be reached. One possible reason for higher‐than‐expected compatibility could be due to the fact that cellulose is inherently partly hydrophobic on one side of its backbone structure [21], and for Na‐CMC with degree of substitution values below 1 (0.9 for this particular Na‐CMC), the charge distribution can also be uneven and patchy [22], which would facilitate attractive interactions with the surfactants on the unsubstituted parts, and possibly appearance of a CAC and thus enhanced compatibility. The shear‐induced birefringence seen for polymer samples at 2 wt% is in this case not caused by wormlike micelles, since it can be seen even at the lowest concentration of the surfactant (which is not prone to micellar growth). Instead, this is most likely caused by the polymer itself aligning in the bulk when sheared. While most of the viscosity is in this case most likely built by the polymer itself, there is still some indications of synergistic effects seen by a slight viscosity maximum around 10–12 wt% C10MT at 1 wt% polymer, and in that the viscosity is higher at the highest surfactant concentrations with 2 wt% polymer.

Although desired compatibility is observed, this is unfortunately disrupted already with 2 wt% added NaCl for both 1 and 2 wt% polymer. In this case, ‘gel blobs’ most likely rich in polymer are formed. It is not entirely clear why they are formed, but most likely the addition of salt reduces the electrostatic repulsions which in turn facilitates the hydrophobic polymer–polymer interactions [22], thus letting the polymer aggregate and gel in lumps, which may or may not also contain surfactant. In fact, the hydrophobic polymer–polymer interactions might even be enhanced by surfactant interactions as the case for associative thickeners such as HM‐HEC in previous section. Finally, at 2 wt% polymer, 5 wt% added salt and 15 wt% C10MT, the sample also separates in a turbid bottom phase containing gel blobs and a clear low viscosity top phase. At this stage, the system has most likely reached true segregative phase separation where polymer and surfactant no longer mix. However, it could also be that once the electrostatic repulsion is reduced by the presence of salt, the hydrophobic interactions remain and eventually dominates actually leading to associative phase separation.

Compatibility was greatly reduced for the system where Na‐CMC was instead combined with AOS, where even salt free samples are turbid at 15 wt% surfactant with 1 wt% polymer, and already at 12 wt% surfactant in the 2 wt% polymer sample. Adding only 1 wt% salt to these samples completely disrupted compatibility, leading to even more turbidity and obvious phase separation as can be seen in Figure 4, Tables 8 and 9.

While this study has focused on the effect of salt, one should also consider the amounts and presence of other substances or impurities introduced together with these newer materials, such as for example surfactant isomers with a resulting change in the so‐called critical packing parameter (CPP) and resulting self‐aggregating behaviour. The presence of these substances, variations or impurities can contribute to incompatibilities, but also in the same way be reasoned around and controlled using understanding of the same fundamental interactions discussed here.

## CONCLUSIONS

This study has shown that many new polymer–surfactant combinations that are first viewed as incompatible when used as received (as previously assessed at Procter & Gamble) can be miscible even at high surfactant concentrations as long as the background salt often included in these newer materials is first removed. Sometimes part of the salt can be added back in a controlled manner, maintaining compatibility. In other words, prior knowledge and theory based on classical polymer surfactant interactions and compatibility still holds, and thus predictions can be made. However, the balance is fine, and it has proven difficult to reach a mixture where compatibility is maintained at a surfactant concentration and viscosity level, which is desired for the specific final formulation. The only combinations where desired conditions were truly met was for 2 wt% Na‐CMC at 10 wt% AOS without added salt. If slightly lower or higher viscosity would be considered one could also include 2 wt% HM‐HEC at 10–12 wt% AOS with an addition of 1 wt% NaCl (medium high viscosity), and 2 wt% Na‐CMC at 10–15 wt% C10MT without added salt (very high viscosity). All systems proved to be sensitive to additions of salt, although for the HM‐HEC—AOS system small additions of salt could be utilized to extend the polymer–surfactant network regime (with increased viscosity) to slightly higher surfactant concentrations.

We recall from the Introduction that the initially tested systems without reduced background salt either phase separated or gave undesired texture, and we have in the present work shown how stable mixtures can be achieved by carefully controlling the salt levels. The impact on the final performance in a hypothetical formulation of a surfactant used at 15 wt% containing 2.5 wt% background salt and a polymer used at 2 wt% with a similar salt content will be affected by the overall salt content and the main contribution will come from the surfactant in this case (0.375 wt%), whereas only a small contribution to the overall salt content will come from the polymer (0.05 wt%); thus, emphasis on any salt removal from the ingredients should clearly be on the former.

It is our conclusion that the highest compatibility for more viscous systems are seen for polymers with moderate hydrophobicity. However, as seen these systems are sensitive to additions of salt and difficult to balance to get the right number of micelles per hydrophobic entity. Traditionally, this is countered by also introducing physical crosslinks, making huge space filling hydrophobically modified polymers such as Aqua SF‐1 (a crosslinked hydrophobically modified polyacrylate). However, these polymers are not biodegradable nor naturally sourced.

There is a pressing need for new materials that enable thickened, water‐based surfactant formulations in particular with sulphate‐free surfactants at high (>10%) concentration to ensure effective lather and cleansing. While purified surfactants are a potential and effective solution, they can be costly, and moreover, synthetic acrylate polymers, though providing thickening, are less sustainable. Consumers, NGOs and regulatory agencies are rightfully and increasingly advocating for natural and sustainable alternatives where an ideal solution will meet product performance with sustainability and cost‐effectiveness. Developing new polymer and surfactant materials that meet these criteria is crucial for the future of this important consumer product industry.

## CONFLICT OF INTEREST STATEMENT

This work has been performed as a collaboration between CR Competence and The Procter & Gamble Company. All authors are 100% paid by either entity, and costs for this work have been shared between both companies.

## Data Availability

The data that support the findings of this study are available from the corresponding author upon reasonable request.
